# Targeting KIR as a novel approach to improve CAR-NK cell function

**DOI:** 10.20517/jtgg.2023.25

**Published:** 2023-12-05

**Authors:** Lara V. Graham, Jack G. Fisher, Salim I. Khakoo, Matthew D. Blunt

**Affiliations:** School of Clinical and Experimental Sciences, University of Southampton, Southampton SO16 6YD, UK

**Keywords:** Killer immunoglobulin-like receptors (KIRs), natural killer cells (NK cells), chimeric antigen receptor (CAR), immunotherapy, CAR-NK cells

## Abstract

Chimeric antigen receptor (CAR) NK cells are demonstrating promising activity in clinical trials and possess a favorable safety profile compared to CAR-T cells. The Killer cell Immunoglobulin-like Receptors (KIR) have a critical role in the control of NK cell function, and recently, this family of activating and inhibitory receptors have been targeted to improve CAR-NK function. These strategies include the utilisation of inhibitory KIR to reduce trogocytosis-associated NK cell fratricide, the downregulation of inhibitory KIR on CAR-NK cells to alleviate HLA mediated suppression, the selection of CAR-NK cell donors enriched for activating KIR, and the use of activating KIR intracellular domains within novel CAR constructs. These pre-clinical studies demonstrate the potential utility of targeting the KIR to improve CAR-NK cell efficacy and patient outcomes.

## Introduction

The recent success of autologous chimeric antigen receptor (CAR) T cells for the treatment of B-cell malignancies has generated huge interest in CAR-based therapies; however, CAR-T cell therapy is associated with severe side effects, including GvHD, cytokine release syndrome (CRS), and neurotoxicity.

To overcome these issues, CAR-natural killer (NK) cells are being explored as an alternative approach due to their improved safety profile, with clinical trials demonstrating an absence of GvHD, CRS, and neurotoxicity to date^[1–3]^. In addition, CAR-NK cells have promise as an allogeneic “off-the-shelf” product that can be given to multiple patients, allowing for reduced production costs and faster delivery of therapy compared to CAR-T cells. There are currently over 30 active clinical trials evaluating CAR-NK cells in cancer, with donor NK cells sourced from cord blood, iPSC, and peripheral blood^[4]^.

## Targeting The Killer Cell Immunoglobulin-Like Receptors

The improvement of CAR-NK cell function and persistence is under intense investigation, and strategies include the expression of non-cleavable CD16, cytokine stimulation, disruption of TGFβ receptor signalling, and disruption of inhibitory immune checkpoints such as NKG2A. One major family of receptors expressed by NK cells that are critical for their activity but have proven hard to target effectively for immunotherapy are the Killer cell Immunoglobulin-like Receptors (KIRs). The inhibitory KIRs have a critical role in controlling NK cell function and education^[5]^. Upon KIR binding with their HLA-A/B/C ligands, inhibition of NK cell activation is mediated via the intracellular immunoreceptor tyrosine-based inhibitory motifs (ITIM)^[5]^. Downregulation of HLA-A/B/C on the surface of cancer cells causes a loss of inhibitory KIR signalling and thereby unleashes NK cell cytotoxicity against target cells, a paradigm known as “missing-self”^[6]^. The clinical impact of this was first shown in patients undergoing haploidentical stem cell transplantation for leukaemia who had HLA-mismatched grafts^[7]^. Therapeutic targeting of individual inhibitory KIR is challenging due to the diversity of KIR at both population and sequence levels. Therefore, a pan-KIR approach targeting KIR2DL3/L2/L1 with the blocking antibody lirilumab has been investigated in detail. However, in clinical trials, it has not been as successful as would have been suggested by pre-clinical data^[8]^. This may be due to the prolonged blockade of NK education signals, limiting the functional capability of NK treated with this antibody^[9]^. Nonetheless, clinical evaluation of lirilumab is still ongoing in multiple cancer types, so this remains a clinical opportunity^[10]^.

**Figure 1:**
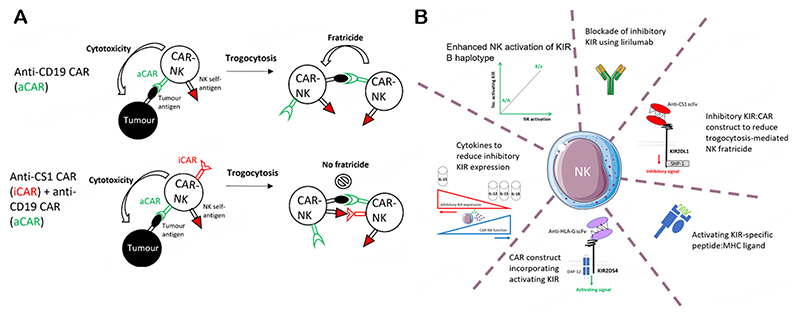
KIR-targeting strategies to improve NK therapy. (A) CAR target tumour antigens can be transferred to CAR-NK cells by trogocytosis^[11]^. Co-expression of a tumour targeting CAR (aCAR) with an inhibitory KIR-based CAR (iCAR) construct recognizing an antigen expressed on NK cells reduces fratricide of CAR-NK cells that are positive for the target tumour antigen due to trogocytosis. (B) Summary of current approaches under investigation targeting the KIR in NK cell therapeutics. Parts of this figure were generated using Servier Medical Art images licensed under a Creative Commons Attribution 3.0 Unported License (https://creativecommons.org/licenses/by/3.0/).

Recently, Li *et al*. performed a series of intricate experiments to demonstrate a novel utility for inhibitory KIR in the potentiation of CAR-NK cell function against cancer via the prevention of trogocytosis-mediated CAR-NK cell fratricide and tumour escape^[11]^. Trogocytosis is the transfer of membrane components from one cell to another^[12,13]^, and the transfer of the CAR cognate antigen from tumour cells to effector cells impedes CAR-T and CAR-NK function^[11,14,15]^. To prevent this, Li *et al*. fused a scFv targeting CS1, which is expressed on all normal NK cells, but not CD19+ tumour cells, with the transmembrane domain and inhibitory domain of the inhibitory KIR, KIR2DL1^[11]^. When this inhibitory anti-CS1 CAR construct was coexpressed with an anti-CD19 CAR on NK cells, cytotoxicity was maintained against CD19+CS1- tumour cells, but cytotoxicity and fratricide against CD19+CS1+ NK cells were inhibited *in vitro* [Figure 1A]. In a murine model of CD19+ lymphoma, NK cells expressing both anti-CS1 CAR and anti-CD19 CAR reduced NK cell fratricide and demonstrated improved efficacy compared to anti-CD19 CAR-NK cells alone. Furthermore, the authors then validated this approach for solid tumours, with enhanced activity evident in a murine model of ovarian cancer for anti-ROR1 CAR-NK cells co-expressing the inhibitory anti-CS1 CAR construct compared to controls.

In summary, Li *et al*. elegantly demonstrate the potent ability of inhibitory KIR mediated signalling to regulate CAR-NK cell function and reveal the impressive utility of inhibitory KIR to improve CAR-NK cell efficacy^[11]^. Although not yet in clinical trials, this approach has strong potential for improving NK persistence and function in patients. This is important because the persistence of adoptively transferred NK cells in patients is currently a key concern for efficacy^[9]^. Furthermore, it would be interesting to assess whether the combination of anti-CS1+anti-CD19 CAR-NK cells can be further improved by combination with cytokine-induced memory-like (ML) NK cells, as anti-CD19-CAR-ML NK cells have recently been shown to possess enhanced efficacy and persistence compared to conventional CAR NK cells *in vitro* and *in vivo*^[16]^.

In addition to the use of inhibitory KIR to impede trogocytosis-mediated fratricide, other approaches targeting the KIR family are being explored [Figure 1B]. These include the use of cytokines to downregulate naturally expressed inhibitory KIR on NK cells and thereby release of CAR-NK cells from inhibitory HLA-A/B/C signals. For example, IL-12, IL-15, and IL-18 cytokine stimulation of NK cells reduced inhibitory KIR expression compared to IL-15 alone^[17]^ and improved anti-CD19 CAR-NK function *in vitro* and *in vivo* compared to CAR-NK cells cultured in IL-15 alone^[18]^. Interestingly, the expression of inhibitory KIR2DL2 has also been reported on CAR-T cells, where KIR2DL2 impaired CAR-T function and CRISPR-Cas9 ablation of KIR2DL2 improved CAR-T function *in vitro* and *in vivo*^[19]^, indicating that an analogous approach may be applicable to CAR-NK cells. An alternative approach that would be of interest to assess is whether the extracellular domain of inhibitory KIR can be fused with an activating transmembrane domain to convert suppressive signals into stimulation and hence overcome immunosuppression via HLA-A/B/C expression on tumour cells. A similar approach has been demonstrated in CAR-T cells, whereby the fusion of the extracellular domain of PD-1 with the intracellular domain of CD28 potentiated CAR T cell function *in vivo*^[20]^.

In contrast to the inhibitory KIR, activating KIRs non-covalently associate with the immunoreceptor tyrosine-based activation motif (ITAM)-containing protein DAP-12, and upon receptor ligation lead to phosphorylation of downstream targets and NK cell activation^[21,22]^. There is substantial population diversity in the inheritance of the KIR genes and expression can be divided into two haplotypes: A and B. Haplotype A contains predominantly inhibitory KIR, whereas haplotype B encodes inhibitory KIR, but also more activating KIR^[5]^. This diversity in the frequency of activating and inhibitory KIRs between individuals has been associated with clinical responses in cancer, with the KIR B haplotype being beneficial in the transplantation setting for haematological malignancies in numerous studies^[23–25]^. These clinical data are supported by functional data demonstrating enhanced activation of NK cells that express activating KIR^[23,26,27]^. These results indicate that NK cells from donors with the activating KIR-rich haplotype B may represent a better source of NK cells for the development of allogeneic NK cell therapy. Indeed, the selection of NK cell donors based on the KIR haplotype B has entered clinical trials (NCT04673617)^[28]^. Whether NK cells that express activating KIR retain their superior activation following clinically relevant *ex vivo* expansion has not been investigated in detail; however, data presented at the Society for Immunotherapy of Cancer (SITC) meeting in 2021 indicate that enhanced cytotoxicity of CAR-NK cells is associated with donors containing a higher number of activating KIR genes when expanded on K562-mbIL15-41BBL stimulatory cells with IL-2, IL-12, and IL-18^[29]^. Interestingly, this enhanced function was not reported in the absence of IL-12 and IL-18 ^[29]^, indicating that the selection of the NK cell expansion strategy must be carefully evaluated to harness the potential benefit of activating KIR in the allogeneic setting.

Further enhancement of CAR-NK through the KIRs may include agonism of activating KIR through monoclonal antibodies or Natural Killer Cell Engagers (NKCEs)^[30]^. However, targeting of activating KIR is hindered by 97%-98% sequence homology between inhibitory and activating KIR in the extracellular domains^[22]^; therefore, designing protein-based reagents capable of distinguishing the different KIRs remains challenging. Peptide:MHC constructs specific to the activating receptor KIR2DS2 have been identified^[31]^, and the introduction of this construct through a DNA therapeutic vaccine approach has shown efficacy *in vivo* in KIR transgenic mice^[32]^. Of note, the specificity of KIR2DS2 for peptide:MHC was found to be unaltered by allelic diversity, suggesting that targeting specific isoforms of KIR2DS2 offers no advantage^[31]^. Testing whether this approach can potentiate CAR-NK function will be an interesting area for future research.

An alternative strategy for utilising the activating KIR was developed by Jan *et al*., whereby the transmembrane and intracellular domains of the activating receptor KIR2DS4 were used for the generation of an anti-HLA-G CAR construct^[33]^. These anti-HLA-G CAR expressing NK cells showed potent cytotoxicity against solid tumour cell lines *in vitro* and human breast cancer cells *in vivo*. The most appropriate intracellular stimulating domains for CAR-NK cell therapy have not yet been established, and this may be context-dependent; however, the use of CAR transmembrane domains typically expressed in NK cells, rather than T cells, has shown promise for enhanced CAR-NK cell function^[34–36]^.

## Conclusion

The activating and inhibitory KIR family are critical regulators of NK cell function; however, the KIR have proven hard to utilise for effective immunotherapeutic strategies to date. Unleashing NK cell inhibition or direct stimulation of activating KIR offers promising approaches for NK cell-based therapeutics. In addition, the application of KIR for CAR-NK cell therapy has opened a new window for their utilisation. Rather than using the ligand binding specificity of KIR, approaches using the inhibitory signalling domains to abrogate undesirable activation signals are proving a novel opportunity for the prevention of NK cell fratricide. This may be critical because the persistence of allogeneic NK cells is currently a major focus to improve efficacy. With the rapid progress of CAR-NK cell clinical development, there is huge potential for targeting KIR to fine-tune NK cell activation and maximise patient benefit.

## Data Availability

Not applicable.
